# Neonatal androgenization of hypogonadal (hpg) male mice does not abolish estradiol-induced FSH production and spermatogenesis

**DOI:** 10.1186/1477-7827-3-48

**Published:** 2005-09-21

**Authors:** Margaret O Nwagwu, Helen Baines, Jeffrey B Kerr, Francis JP Ebling

**Affiliations:** 1 School of Biomedical Sciences, University of Nottingham Medical School, Queen's Medical Centre, Nottingham NG7 2UH, UK; 2 Department of Anatomy and Cell Biology, Monash University, Victoria 3800, Australia

## Abstract

**Background:**

Testicular development is arrested in the hypogonadal (hpg) mouse due to a congenital deficiency in hypothalamic gonadotropin-releasing hormone (GnRH) synthesis. Chronic treatment of male hpg mice with estradiol induces FSH synthesis and secretion, and causes testicular maturation and qualitatively normal spermatogenesis. As estradiol negative feedback normally inhibits FSH production in the male, this study tested whether this paradoxical response to estradiol in the male hpg mouse might be due to inadequate masculinisation or incomplete defeminization in the neonatal period. Previous studies have demonstrated that treatment of hpg mice with testosterone propionate in the immediate neonatal period is necessary to allow full reproductive behaviors to be expressed following suitable endocrine stimulation at adult ages.

**Methods:**

Hpg mice were treated with 100 μg testosterone propionate or vehicle on postnatal day 2. At 35 days of age, subgroups of these mice were treated with silastic implants containing estradiol or cholesterol. Reproductive behavior was scored in tests with steroid-primed female mice, then testicular development was assessed histologically, and measures of pituitary FSH content made at 85 days of age.

**Results:**

The neonatal testosterone propionate treatment successfully defeminized female litter mates, as revealed by impaired vaginal opening and deficiencies in lordosis behavior, and it allowed appropriate male reproductive behavior to be expressed in a proportion of the hpg males when tested at an adult age. However, neonatal androgen supplementation did not block or even reduce the subsequent actions of estradiol in increasing pituitary FSH content, nor did it affect the ability of estradiol to induce qualitatively normal spermatogenesis.

**Conclusion:**

The ability of the hpg male to show a "female" neuroendocrine response to estradiol is not a result of inadequate androgenization during neonatal development, and thus the actions of estradiol revealed in this rodent model are not an artefact of incomplete sexual differentiation, but reflect a physiological role of estradiol occurring during a specific early temporal window of male reproductive development.

## Introduction

Although estradiol has classically been considered a female hormone, recent data from man shows that it plays important physiological roles in the male. For example, estradiol deficiency or resistance results in lack of bone epiphyseal fusion, delayed skeletal maturation and low sperm viability [[1] for review]. These effects can be reproduced in rodent models so that the underlying mechanisms of estrogen action can be investigated. For example, male mice in which estrogen receptor (ER) α has been knocked out become progressively infertile [2,3], and likewise, if production of estradiol is prevented by knockout of the cyp19 aromatase gene, then such mice show impaired spermatogenesis, reduced spermatid numbers and infertility [4].

We have used the hypogonadal (*hpg*) mouse to study the actions of estradiol in male reproduction. Such mice are unable to produce gonadotrophin releasing hormone (GnRH) due to a truncation in the GnRH gene, and therefore show a profound hypogonadotrophic hypogonadism [[5] for review]. Surprisingly, treatment of *hpg *males with low levels of estradiol stimulates spermatogenesis, as evident by an increase in testis weight and the presence of elongated spermatids in the seminiferous tubules of the testis [6]. This induction of spermatogenesis is accompanied by increases in pituitary FSH content and in circulating FSH concentrations [6,7]. FSH is an important component of the spermatogenic process; lack of the FSH β subunit or receptor in genetically-modified mice results in decreased testis size and reduced sperm quality [8]. Conversely, treatment of *hpg *mice with recombinant human FSH has been shown to increase testis size and the number of spermatogonia [9].

In male mammals, estradiol normally provides a negative feedback signal which inhibits FSH synthesis and secretion [6,10]. Thus, the increase in FSH production in response to estradiol in *hpg *mice ("positive feedback") might be considered to be a "female" neuroendocrine response. One possibility is that the phenomenon of estradiol-induced FSH production in male *hpg *mice is due to inadequate masculinization or incomplete defeminization of the neonate due to the lack of androgen exposure in the early postnatal period. Appropriate pre- and postnatal testosterone concentrations are known to be necessary for complete masculinization in rodents. Before postnatal day 5, serum testosterone is higher in male mice compared to females [11] and this difference is believed to be important for defeminization of males. Mice and other rodents have a critical period of neural sexual differentiation before postnatal day 10. Experimental studies have demonstrated that administration of testosterone to female mice in early postnatal life suppresses sexual receptivity and increases aggression at later ages [12]. Conversely, neonatal castration of male mice results in a lack of normal male aggressive behavior in adulthood; which is not restored with later testosterone treatment [13]. Hence early androgen exposure serves to differentiate the subsequent propensity to display aggressive behavior and sexual receptivity [13], and sensitizes appropriate neural elements to androgens encountered in later life [12].

There is clear evidence that *hpg *mice are not adequately masculinized in the neonatal period. *Hpg *males will show appropriate *physiological *reproductive development when treated in adulthood with either grafts of fetal hypothalamic tissue releasing GnRH or with appropriate gonadotropins [5], but importantly such *hpg *males do not show appropriate reproductive *behavior *despite the induction of steroidogenesis and spermatogenesis. However, if *hpg *mice are also treated with exogenous androgens on postnatal day 2, and then the reproductive axis is activated by grafts or gonadotropins, they subsequently display mounting, intromission and ejaculation, and can sire litters in later life [14]. Therefore, the aim of this study was to test the hypothesis that the ability of estradiol to increase in FSH production in male *hpg *mice is due to the lack of defeminization or masculinization resulting from low testosterone exposure of *hpg *male mice during the early postnatal period.

The experimental approach was to administer testosterone propionate to neonatal male *hpg *mice. We predicted that if the ability to respond to estradiol is a consequence of an inadequate postnatal androgen environment in *hpg *mice, then neonatal treatment with testosterone propionate should block the ability of subsequent estradiol treatment to produce a rise in pituitary FSH and to induce spermatogenesis.

## Materials and methods

### Animals

**Figure 1:**
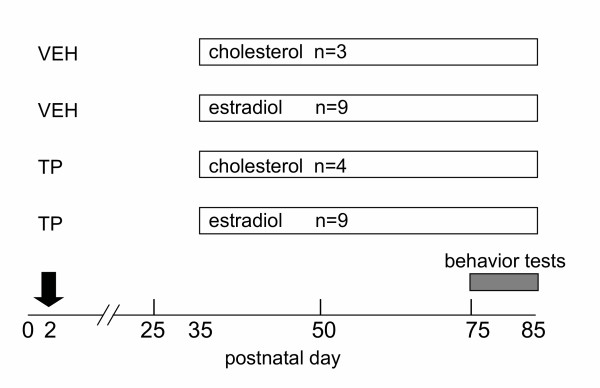
Experimental design. Male *hpg *mice were treated with either vehicle (VEH) or 100 μg testosterone propionate (TP) on postnatal day 2, then received a subcutaneous silastic capsule containing either cholesterol (chol) or 2% estradiol in cholesterol on day 35 of age.

All animal procedures were approved by the University of Nottingham Local Ethical Review Committee and carried out in accordance with the Animals Scientific Procedures Act (UK) 1986 (project licence PPL 40/2372). Laboratory Animal Science Association (LASA) guidelines were followed for administration of substances [15]. Male and female mice known to both be heterozygous for the *hpg *mutation as determined by PCR-based genotyping [16] were housed in breeding cages (n = 15 pairs). All male (n = 126) and female (n = 129) pups born to these breeding pairs were treated with testosterone propionate (100 μg sc) or vehicle alone (arachis oil) on postnatal day 2 (Figure 1). On postnatal day 35, male *hpg *mice (n = 25) were identified by their micropenis and small scrotal sac, and were then given a subcutaneous (sc) implant containing 2% estradiol (n = 18) or cholesterol (n = 7) as previously described [6]. Implants were left in place for 50 days, about 1.5 spermatogenic cycles (Figure 1).

### Sexual behavior: male hpg mice

Sexual behavior was assessed in *hpg *males a few days prior to the end of the study (Figure 1). The steroid priming and testing procedure was adapted from the method of McGill [17]. Wild-Type adult C3H female mice were brought in to estrous by treatment with 35 μg of estradiol benzoate followed 36–48 hours later by injection of 100 μg progesterone, and testing was conducted 6 hours following progesterone treatment. These females were initially tested with proven male studs in order to confirm that they were sexually receptive. For the test, *hpg *males or studs were placed in an observation cage 48 hours prior to testing in order to ensure adaptation to the new cages. Males were tested on several occasions with at least two receptive females; test(s) were carried out by placing a sexually receptive female into the male cage for 15 minutes, and the number of mounts and intromissions observed and recorded.

### Female sexual behavior

Female C3H mice injected with vehicle or testosterone propionate on postnatal day 2 were weighed weekly after weaning and were scored for vaginal opening every week until day 70 of age. These females were tested on consecutive days with male studs as described above and were observed to see if they were receptive (e.g. exhibiting lordosis) or whether they persistently non-receptive i.e. demonstrating aggressive behavior by attacking or chasing males round the cage.

### Hormone measurements and testis collection

On postnatal day 85, the experimental males and three age-matched wild-type litter mates were killed by anesthesia overdose (sodium pentobarbitone, Rhone Merieux, Harlow). The pituitary, testes, epididymides and seminal vesicles were excised, trimmed of fat and connective tissue, and weighed. One testis was placed in Bouin's fixative and the other was placed on ice and frozen at -20°C. The fixed testes were processed into paraffin blocks and 5 μm sections were stained with haematoxylin-eosin for histological analysis. For measurement of testis testosterone content, the frozen testis was sonicated in 1 ml PBS (3 × 10 seconds) and the homogenate extracted twice with 4 ml of diethyl-ether. The supernatant was left to evaporate overnight in a fume hood at 25°C, and samples were reconstituted in 1 ml PBS. Testosterone was measured using a salivary testosterone ELISA assay kit (IDS Ltd., Tyne & Wear, UK). The minimum detection limit was 0.006 ng/well. Pituitary glands were sonicated in 500 μl PBS and FSH content was measured in a single assay using a commercially available Rat FSH IRMA kit (IDS Ltd., UK). The minimum detection limit was 0.2 ng/tube and the intra-assay CV was 1.8%.

### Histological examination of fixed testis

Three sections from each of 3–5 fixed testes from each treatment group were examined. The sections were scored for the presence of lumina and elongating spermatids by an observer who was blind to the experimental treatment. Tissue sections with no evidence of lumen formation in any tubules received a score of 0, sections where less than 50% of tubules containing a lumen received a score of 1, sections where less than 50–95% of tubules had lumina received a score of 2, and sections where all tubules contained a lumen received a score of 3. A similar scoring system was used to estimate the prevalence of elongating spermatids. The mean score for each testis was then calculated, and then used to calculate the group mean score.

### Statistical analysis

All statistical analysis was performed using Prism v3 (GraphPad Software, San Diego, CA). Results were analysed by t-test, Chi-squared tests, 2 factor ANOVA or Kruskal-Wallis tests as appropriate.

## Results

### Effect of neonatal androgenization on development and behavior of female littermates

**Figure 2:**
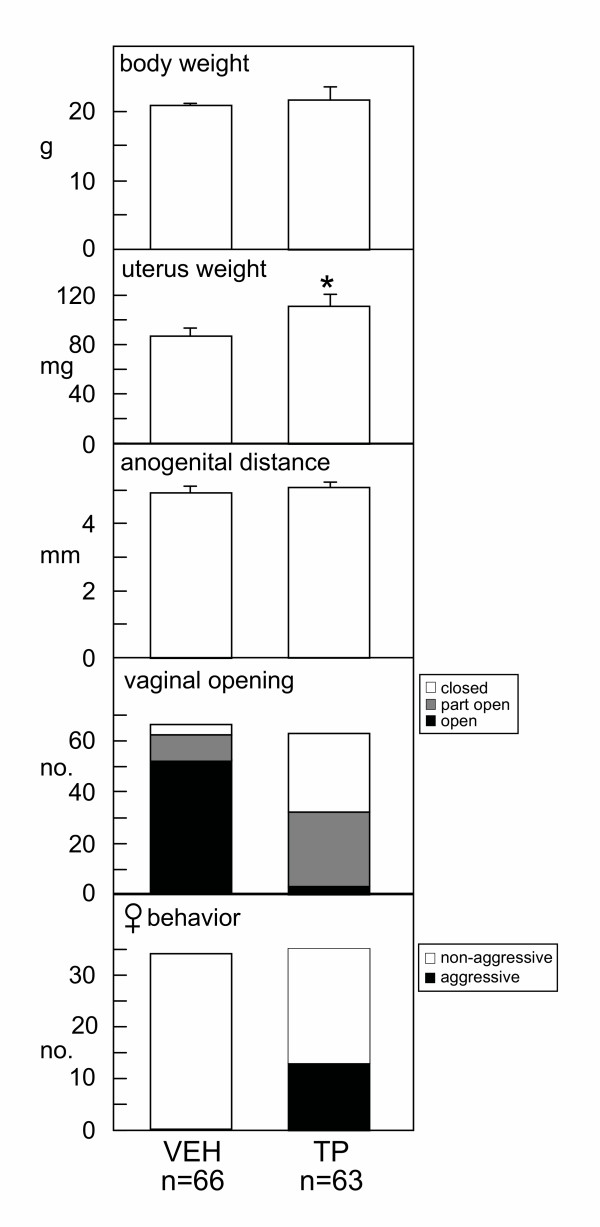
Female littermates of experimental male mice received a subcutaneous injection of either vehicle (VEH) or 100 μg testosterone propionate (TP) on postnatal day 2. Panels indicate body weight (top), reproductive tract weight, anogenital distance, proportion showing vaginal opening by day 70 of age and proportion showing aggressive behavior toward stud males in tests of reproductive receptivity. Values are group mean ± SE (upper panels) or numbers of mice (lower panels).

Vaginal opening and sexual behavior were scored in female litter mates to confirm the validity of the androgenization protocol. The mean weight of the uterus in 70 day old female mice treated neonatally with testosterone propionate was significantly (p < 0.05) greater than that of vehicle-treated females (Figure 2). There was no significant difference in the anogenital distance of the two groups (Figure 2, middle panel), but Chi-squared tests confirmed a significant (p < 0.05) reduction in the proportion of females with full vaginal opening (3% after neonatal testosterone propionate treatment compared to 79% after vehicle treatment, Figure 2). In addition, 37% (13 of 35) of female mice treated neonatally with testosterone propionate showed atypical aggressive behavior and attacked stud males, but this behavior was never observed in any vehicle-treated females (Figure 2, bottom panel, P < 0.05; Chi-squared).

### Effect of neonatal androgenization on sexual behavior in adult hpg males

**Figure 3:**
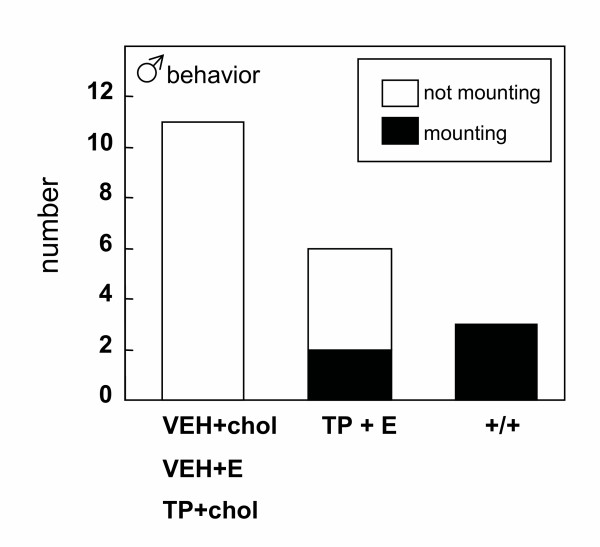
Proportions of male *hpg *mice displaying mounting behavior when paired with a steroid-primed female. Mice were treated with vehicle (VEH) or testosterone propionate (TP) on postnatal day 2, then either a cholesterol (chol) or a 2% estradiol (E) subcutaneous implant on day 35, and tested after 75 days of age. Three wild-type C3H mice were also tested (+/+).

Chi-squared tests revealed a significant (p < 0.05) difference in the proportion of males displaying mounting behavior. No mounting behavior was ever observed in *hpg *males treated with vehicle in the neonatal period and with either cholesterol or estradiol implants from day 35, or treated with testosterone propionate neonatally and then with cholesterol implants from day 35. However, 2 of 6 (33%) of *hpg *mice treated neonatally with testosterone propionate and subsequently with estradiol implants from day 35 demonstrated mounting behavior when tested after a further 40 days (Figure 3). All the wild-type studs were also observed to mount steroid-primed females (Figure 3).

### Effect of neonatal androgenization on estrogen-induced spermatogenesis and FSH production in hpg males

**Figure 4:**
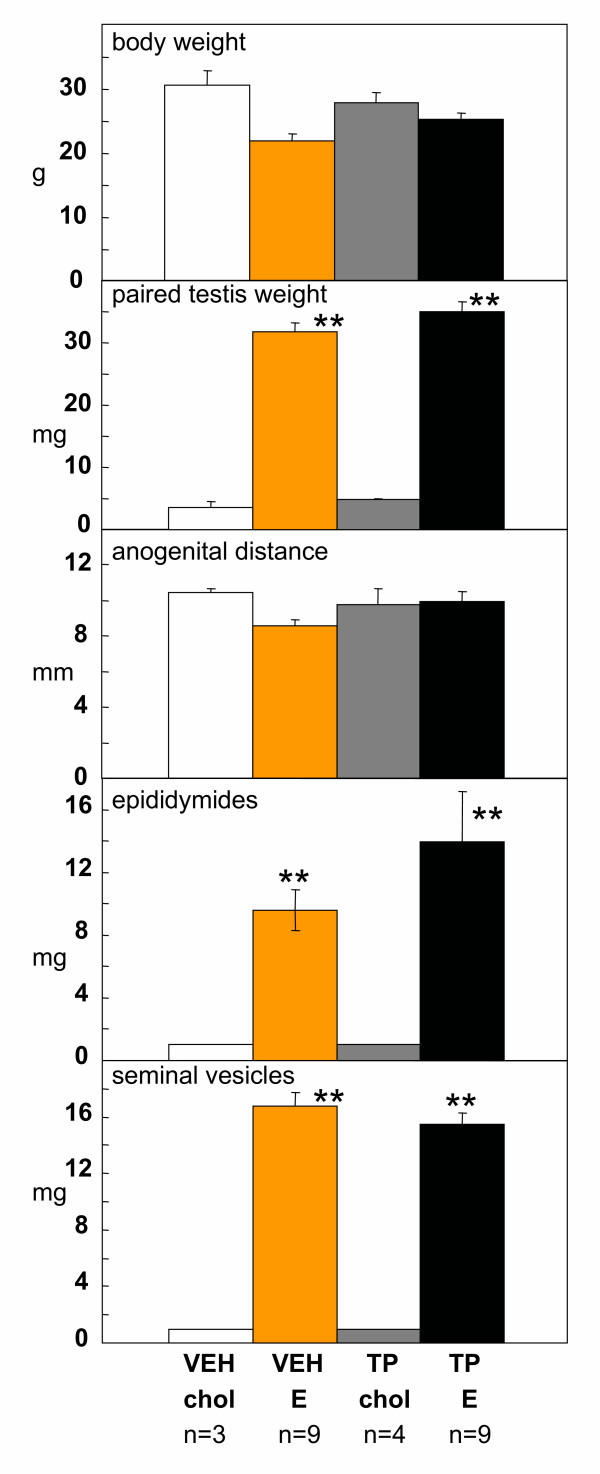
Body weight (top), paired testis weight, anogenital distance, and wet weight of epididymides and seminal vesicles (bottom)) of male *hpg *mice receiving vehicle (VEH) or 100 μg testosterone propionate (TP) on postnatal day 2, then either a cholesterol (chol) or a 2% estradiol (E) subcutaneous implant on day 35. Values are group mean ± SE. **P < 0.001 vs groups treated with cholesterol implants.

**Figure 5:**
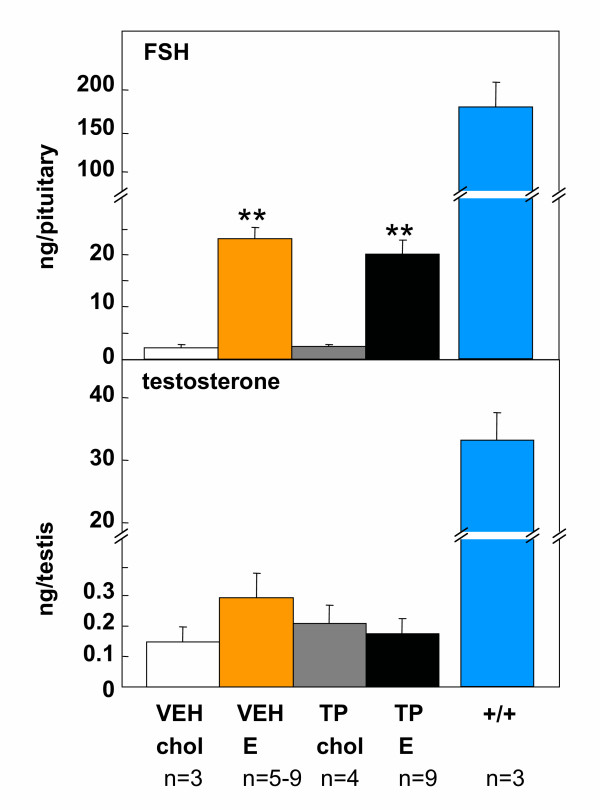
Pituitary FSH content (top) and testis testosterone content (bottom) in male *hpg *mice; receiving either vehicle (VEH) or testosterone propionate (TP) on postnatal day 2, then either a cholesterol (chol) or a 2% estradiol (E) subcutaneous implant on day 35. For comparison, pituitary FSH and testicular testosterone values derived from wild-type litter mates analysed in the same assay are indicated (+/+). Values are group mean ± SE. **P < 0.001 vs groups treated with cholesterol implants.

In male *hpg *mice, neonatal androgenization *per se *had no significant effect on body weight (Figure 4, top), testis weight (Figure 4) or anogenital distance (Figure 4). Treatment with estradiol significantly increased the weights of the testes, epididymides and seminal vesicles (Figure 4) (p < 0.05), regardless of whether the mice had been treated neonatally with testosterone propionate or vehicle. Estradiol treatment also significantly (p < 0.05) increased pituitary FSH content in *hpg *mice that received either testosterone propionate or vehicle on postnatal day 2 (Figure 5 upper panel). Two-factor ANOVA using the values for organ weights and FSH content revealed no significant interactions between neonatal treatment (testosterone propionate vs vehicle) and subsequent treatment (estradiol vs cholesterol implant), thus neonatal androgenization did not influence the subsequent response to estradiol treatment. Testicular testosterone content was not significantly affected by neonatal androgenization or subsequent estradiol treatment (Figure 5 lower panel).

### Histological analysis

**Figure 6:**
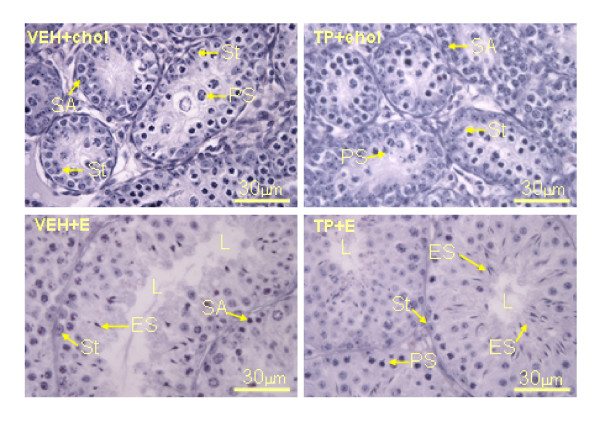
Representative examples of testicular histology from 85 day old *hpg *mice treated with vehicle (VEH) or testosterone propionate (TP) on postnatal day 2, then receiving either a cholesterol (chol) or a 2% estradiol (E) subcutaneous implant on day 35. Note that the most mature cells types in testes from mice treated with cholesterol (VEH+chol, TP+chol) are type A spermatogonia (SA) and primary spermatocytes (PS); there is little evidence of lumen formation, and Sertoli cells are frequently observed adjacent to the basal lamina and more centrally (St). In contrast, after estradiol treatment (VEH+E, TP+E) the seminiferous tubules are expanded and have developed a lumen (L), Sertoli cells are adjacent to the basal lamina and round and elongating spermatids (ES) are present. Treatment with testosterone propionate in the neonatal period did not affect the testicular response to estradiol in later life. Scale bars represent 30 μm.

The mean scores for the histological examination of testes are shown in Table 1. Testes from *hpg *mice that only received cholesterol implants in adult life had a characteristic undeveloped appearance, regardless of whether the mice had received testosterone propionate or vehicle during the neonatal period. The seminiferous tubules were of small diameter (Figure 6), generally lacking a lumen (Figure 6, Table 1), with Sertoli cells frequently located medial to the basal lamina (Figure 6). Round and elongating spermatids were not observed, the most mature cells types in testes from *hpg *mice treated with cholesterol were spermatogonia type A and pre-pachytene primary spermatocytes (Figure 6). In contrast, after estradiol treatment the seminiferous tubules had expanded and developed a lumen, regardless of whether the mice had been treated with testosterone propionate or vehicle neonatally (Figure 6, Table 1). Sertoli cells were observed to be adjacent to the basal lamina, and round and elongating spermatids (ES) were present (Figure 6). Thus, treatment with testosterone propionate in the neonatal period did not affect testicular histology after treatment with estradiol in later life.

**Table 1:** 

	VEH+chol	VEH+E	TP+chol	TP+E
lumen	0.7 ± 0.3	2.8 ± 0.2	0.3 ± 0.3	3.0 ± 0.0
elongating spermatids	0.0 ± 0.0	2.2 ± 0.5	0.0 ± 0.0	2.3 ± 0.7

Assessment of the presence of lumen and elongating spermatids. Mice were treated with vehicle (VEH) or testosterone propionate (TP) on postnatal day 2, then either a cholesterol (chol) or a 2% estradiol (E) subcutaneous implant on day 35, and testes tested at 85 days of age. Values (from 0 to 3, see text) are group mean scores ± SE of 3–5 testes per treatment group.

## Discussion

The principal findings of this study were firstly that all the *hpg *male mice treated with estradiol demonstrated clear increases in pituitary FSH content and in the wet weight of the testes, epididymides and seminal vesicles, reflecting activation of spermatogenesis as evidenced by the presence of elongated spermatids in the seminiferous tubules. This paradoxical stimulatory action of estradiol in the male is in agreement with our previous studies in the *hpg *mouse [6,7] and confirms the robustness of the response. Secondly, and more importantly, androgenization of *hpg *males with testosterone propionate in the neonatal period did not affect the subsequent ability of estradiol to activate the reproductive axis in such mice.

Since the estradiol-induced rise in pituitary FSH was not abolished by neonatal androgenization of *hpg *male mice, the possibility arises that the current neonatal androgenization protocol was ineffective in the induction of masculinization of the *hpg *males. However, we followed carefully a well-established dosing protocol previously demonstrated to be effective in male *hpg *mice [14], and our other experimental observations suggest the androgenization protocol was at least partly successful. The neonatal testosterone propionate injections were certainly effective in defeminizing the female litter mates of the *hpg *males. First, vaginal opening usually occurs around postnatal day 35 in female mice [18], yet the vast majority of our androgenized females (97%) failed to show full vaginal opening by 70 days of age. Second, a significant proportion (36%) of androgen-treated females displayed aggressive behavior when paired with stud male mice, consistent with previous studies demonstrating that increased aggression is a consequence of neonatal androgenization in female mice [13]. Third, the uterine weights from the females that were treated with testosterone propionate in the neonatal period were significantly greater than those from the vehicle-treated females, also consistent with previous studies demonstrating that androgenized female mice have heavier uteri [19]. The observation that vaginal opening and uterine weight were affected in almost all neonatally-androgenized females whereas a lower proportion displayed abnormal female behavior may indicate that a higher androgen threshold must be reached to affect sexual behavior than to disrupt the endocrine control of the female reproductive tract.

There was also evidence of masculinization of behavior in some of the *hpg *males: two of the six *hpg *males treated neonatally with testosterone propionate and then with estradiol in later life displayed mounting and intromission when paired with sexually receptive female mice. Such behaviors did not occur in the *hpg *mice treated with vehicle in the neonatal period, consistent with the previously hypothesised crucial role of early androgen exposure in mice for masculinizing the brain and the sexually dimorphic spinal nucleus of the bulbocavernosus and its target perineal muscles, which are involved in the control of penile copulatory reflexes [14,20,21]. The failure of the other four neonatally-androgenized mice to display copulatory behaviors is most likely to relate to the limiting endocrine milieu at the time of the behavioural tests. Previous studies of the role of neonatal androgens in regulating male reproductive behavior in *hpg *mice relied upon additional treatment with testosterone propionate in adult life to permit such behaviors to be expressed in an appropriate context [14]. In the current experiment, levels of intratesticular androgens were found to be low even after estradiol treatment, and our previous studies have likewise failed to detect rises in circulating androgen concentrations after estradiol treatment [6]. Thus, the prevailing androgen milieu is very likely to be inadequate for most mice to be primed to display mounting, intromission and ejaculation.

In view of the evidence of successful androgenization in at least some of the *hpg *males and female siblings, our interpretation of the current studies is that the increase pituitary FSH content in male *hpg *mice in response to estradiol is *not *a failure of early masculinization or defeminization. An alternative explanation may be that the positive FSH response of gonadotrophs to estradiol in "adult" male *hpg *mice may be due to the immaturity of the pituitary gland reflecting the lack of pituitary exposure to GnRH in such mice. A recent study of the expression of the pituitary melatonin receptor 1 (MT1) gene provides good evidence that the pituitary gland in *hpg *mice is arrested at an immature stage of development [22]. In normally developing mice exposed to increasing amounts of GnRH, there is a decline in MT1 expression in the pituitary gland, but in *hpg *mice which are not exposed to GnRH, this decline does not occur [22]. Developmental changes in gonadotroph secretory profiles have been described for rats [23] and rhesus monkeys [24], so it might be hypothesized that ability of estradiol to increase synthesis and secretion of FSH (but not LH, 7) in *hpg *mice is a reflection of an action of estradiol that is physiologically relevant during early postnatal development in mammals. Indeed, stimulatory actions of estradiol on testis function have been demonstrated in several other rodent models where pituitary development is still immature, and provide some evidence that estradiol exerts its stimulatory effects via an interaction with FSH. For example, treatment of rats with estradiol in the second postnatal week of life stimulates germ cell mitosis increasing the abundance of type A spermatogonia [25]. Correspondingly, treatment of neonatal rats with diethylstilbestrol induces small increases in circulating FSH and advances the initiation of spermatogenesis at puberty [26]. However, these effects of estradiol may not be exclusively via action on pituitary gonadotrophs producing FSH, because in a similar experimental paradigm in neonatal rats, estradiol treatment also enhances the actions of FSH within the seminiferous tubule on pre-meiotic differentiation, resulting in increased abundance of pachytene spermatocytes [27].

## Conclusion

Treatment of male *hpg *mice with physiological levels of estradiol promotes production of FSH in the pituitary gland and induces spermatogenesis. We hypothesized that this paradoxical response to estradiol might be the result of inadequate masculinisation or incomplete defeminization of the *hpg *male, but this seems unlikely since treatment of neonatal *hpg *mice with testosterone propionate does not abolish these effects of estradiol. The stimulatory responses to estradiol revealed in *hpg *mice at an adult age probably mimic physiological actions of estradiol in early male development when pituitary maturation is incomplete.

## Authors' contributions

MON, HB, JBK and FJPE all participated in the experimental design, MON, HB and FJPE carried out the experimental procedures, MON collated the results and provided a first draft of the manuscript, JBK and FJPE assisted with analysis of the data and completed the writing of the manuscript.
